# ^68^Ga-FAPI-PET/CT in patients with various gynecological malignancies

**DOI:** 10.1007/s00259-021-05378-0

**Published:** 2021-05-29

**Authors:** Katharina Dendl, Stefan A. Koerber, Rebecca Finck, Kgomotso M. G. Mokoala, Fabian Staudinger, Lisa Schillings, Ulrike Heger, Manuel Röhrich, Clemens Kratochwil, Mike Sathekge, Dirk Jäger, Jürgen Debus, Uwe Haberkorn, Frederik L. Giesel

**Affiliations:** 1 Department of Nuclear Medicine, Heidelberg University Hospital, Heidelberg, Germany; 2 Department of Radiation Oncology, Heidelberg University Hospital, Heidelberg, Germany; 3 Heidelberg Institute of Radiation Oncology (HIRO), Heidelberg, Germany; 4 National Center for Tumor diseases (NCT), Heidelberg, Germany; 5 Department of Nuclear Medicine, University of Pretoria & Steve Biko Academic Hospital, Private Bag X169, Pretoria, 0001 South Africa; 6 Department of General, Visceral and Transplantation Surgery, University of Heidelberg, Heidelberg, Germany; 7 Department of Medical Oncology, Heidelberg University Hospital and National Center for Tumor Diseases (NCT), Heidelberg, Germany; 8 Heidelberg Ion-Beam Therapy Center (HIT), Department of Radiation Oncology, Heidelberg University Hospital, Heidelberg, Germany; 9 German Cancer Consortium (DKTK), partner site, Heidelberg, Germany; 10 Clinical Cooperation Unit Radiation Oncology, German Cancer Research Center (DKFZ), Heidelberg, Germany; 11 Clinical Cooperation Unit Nuclear Medicine, German Cancer Research Center (DKFZ), Heidelberg, Germany; 12 Translational Lung Research Center Heidelberg (TLRC), German Center for Lung Research (DZL), Heidelberg, Germany; 13 Department of Nuclear Medicine, University Hospital Duesseldorf, Duesseldorf, Germany

**Keywords:** FAPI, PET, Gynecological malignancies, Fibroblast activation protein, SUV

## Abstract

**Purpose:**

^68^Ga-FAPI (fibroblast activation protein inhibitor) is a novel and highly promising radiotracer for PET/CT imaging. The aim of this retrospective analysis is to explore the potential of FAPI-PET/CT in gynecological malignancies. We assessed biodistribution, tumor uptake, and the influence of pre- or postmenopausal status on tracer accumulation in hormone-sensitive organs. Furthermore, a comparison with the current standard oncological tracer ^18^F-FDG was performed in selected cases.

**Patients and methods:**

A total of 31 patients (median age 59.5) from two centers with several gynecological tumors (breast cancer; ovarian cancer; cervical cancer; endometrial cancer; leiomyosarcoma of the uterus; tubal cancer) underwent ^68^Ga-FAPI-PET/CT. Out of 31 patients, 10 received an additional ^18^F-FDG scan within a median time interval of 12.5 days (range 1–76). Tracer uptake was quantified by standardized uptake values (SUV)max and (SUV)mean, and tumor-to-background ratio (TBR) was calculated (SUVmax tumor/ SUVmean organ). Moreover, a second cohort of 167 female patients with different malignancies was analyzed regarding their FAPI uptake in normal hormone-responsive organs: endometrium (*n* = 128), ovary (*n* = 64), and breast (*n* = 147). These patients were categorized by age as premenopausal (<35 years; *n* = 12), postmenopausal (>65 years; *n* = 68), and unknown menstrual status (35–65 years; *n* = 87), followed by an analysis of FAPI uptake of the pre- and postmenopausal group.

**Results:**

In 8 out of 31 patients, the primary tumor was present, and all 31 patients showed lesions suspicious for metastasis (*n* = 81) demonstrating a high mean SUVmax in both the primary (SUVmax 11.6) and metastatic lesions (SUVmax 9.7). TBR was significantly higher in ^68^Ga-FAPI compared to ^18^F-FDG for distant metastases (13.0 vs. 5.7; *p* = 0.047) and by trend for regional lymph node metastases (31.9 vs 27.3; *p* = 0.6). Biodistribution of ^68^Ga-FAPI-PET/CT presented significantly lower uptake or no significant differences in 15 out of 16 organs, compared to ^18^F-FDG-PET/CT. The highest uptake of all primary lesions was obtained in endometrial carcinomas (mean SUVmax 18.4), followed by cervical carcinomas (mean SUVmax 15.22). In the second cohort, uptake in premenopausal patients differed significantly from postmenopausal patients in endometrium (11.7 vs 3.9; *p* < 0.0001) and breast (1.8 vs 1.0; *p* = 0.004), whereas no significant difference concerning ovaries (2.8 vs 1.6; *p* = 0.141) was observed.

**Conclusion:**

Due to high tracer uptake resulting in sharp contrasts in primary and metastatic lesions and higher TBRs than ^18^F-FDG-PET/CT, ^68^Ga-FAPI-PET/CT presents a promising imaging method for staging and follow-up of gynecological tumors. The presence or absence of the menstrual cycle seems to correlate with FAPI accumulation in the normal endometrium and breast. This first investigation of FAP ligands in gynecological tumor entities supports clinical application and further research in this field.

**Supplementary Information:**

The online version contains supplementary material available at 10.1007/s00259-021-05378-0.

## Introduction

Regarding all cancer entities in females, breast cancer accounts for approximately 276.000 (30%) of all estimated new cases in the USA in 2020. Moreover, breast (15%), ovary (5%), and uterine corpus cancer (4%) are among the six most frequent cancer-associated deaths [1]. Therefore, a reliable and precise staging tool is of essence. The current most frequently used diagnostical radiotracer for PET/CT regarding oncological malignancies is ^18^F-FDG, which accumulates in glucose-consuming tissues. Therefore, the accumulation of ^18^F-FDG is influenced by movement, nutrition, and blood glucose levels. Beyond that, it is limited by high physiological background activity in several organs, low glucose transporter, and hexokinase activity in some malignancies as well as its imprecise differentiation between cancerous growths and acute inflammation and thus its missing specificity [2, 3]. Particularly in gynecological malignancies, several additional pitfalls are commonly encountered, such as potentially false-positive uptake of ^18^F -FDG by the endometrium and ovaries in premenopausal patients as well as physiologic accumulation in several benign diseases including uterine fibroids and benign endometriotic cysts [4]. A novel class of radiotracers, subsumed under the term fibroblast activation protein inhibitor (FAPI), demonstrated highly promising results in previously conducted studies regarding various tumor entities. Fibroblast activation protein (FAP) is a type II serine protease belonging to the dipeptidyl peptidase 4 family with both post-proline dipeptidyl peptidase and endopeptidase activity [5]. Furthermore, FAP is expressed by cancer-associated fibroblasts (CAFs), which are part of the stroma in many tumors promoting cancerous growth and are associated with poor prognosis [6, 7]. FAP is also overexpressed in 90% of all epithelial carcinomas, in normal tissue during wound healing, and selectively in benign diseases [5, 8, 9]. With respect to its limitations, ^68^Ga-FAPI-PET/CT might supplement or even be superior to ^18^F-FDG-PET/CT in gynecological tumors and in several malignant and nonmalignant conditions. We aimed to evaluate the impact of ^68^Ga-FAPI-PET/CT in gynecological patients with various malignancies.

## Materials and methods

### Patient cohort

We retrospectively analyzed two cohorts of patients regarding different characteristics. The first group consisted of 31 female patients with different gynecological tumor entities from two centers (University Hospital Heidelberg *n* = 29, University Hospital Pretoria *n* = 2). Data were evaluated in Heidelberg. Out of these 31 patients, 10 additionally received ^18^F-FDG-PET/CT scan which was performed as standard of care for various oncologic indications and not specifically in terms of comparison. However, the median time interval between these scans was only 12.5 days. As we analyzed the data retrospectively, a comparison between FAPI and FDG of all 31 patients was not possible. All patients were referred by their attending oncologist or radiation oncologist in addition to standard diagnostic imaging. Indications were, among others, inconclusive findings, insufficient tumor delineation, or evaluation of last-line experimental FAP-radioligand therapy. Further information on clinical characteristics regarding our cohort were gathered with the help of electronic patient records. Eight out of the 31 gynecological patients were already published by Giesel et al. comparing intra-individually biodistribution and tumor uptake of ^68^Ga-FAPI and ^18^F-FDG PET/CT in patients with various tumor entities and one by Koerber et al [10, 11]. The second cohort comprised 167 female patients with various tumor entities (head-and-neck cancer, lung cancer, pancreatic cancer, gastrointestinal cancer, others). In this cohort, we conducted further uptake evaluation (SUVmean, SUVmax) of the endometrium, breast, and ovary. Subsequently, we categorized this cohort by age as premenopausal (*n* = 12; <35 years), postmenopausal (*n* = 68; <65 years), and unknown menstrual status (35–65 years; *n* = 87) and compared the uptake of the normal hormone-responsive organs. Several patients of this cohort were already investigated under a different scope, with regard to their malignancies, however, not in terms of benign physiologic uptake in hormon-responsive organs such as endometrium, breast and ovary [10, 12, 14, 15]. All patients gave written informed consent to undergo ^68^Ga-FAPI-PET/CT on an individual patient basis following national regulations and the Declaration of Helsinki. The radiopharmaceutical was synthesized and labeled according to the German Pharmaceutical Act §13(2b). The retrospective evaluation of data was approved by the ethics committee of Heidelberg University (S016/2018, S115/2020) and by the ethics committee of the University of Pretoria (permit 881/2019).

### Radiopharmaceuticals and (^68^Ga-FAPI) PET/CT imaging

Imaging data was acquired 60 min after tracer application using two different PET/CT systems (Supplement Table 1) and reconstructed obtaining whole-body images for both ^18^F-FDG and ^68^Ga-FAPI. All PET scans were acquired in 3D mode with an acquisition time of 3–5 min/bed position at both sites. All patients were monitored regarding any new symptoms or abnormalities up to 30 min after the end of the examination. The median time interval between (^18^F-FDG) PET/CT and (^68^Ga-FAPI) PET/CT patients (*n* = 10) was 12.5 days (range 1–89 days). The scans of these 10 patients receiving ^68^Ga-FAPI- PET/CT and ^18^F-FDG-PET/CT were compared based on biodistribution, tumor uptake, and TBRs.

^*18*^*F-FDG imaging:* All patients were told to fast for at least 6 h before the scan. Blood glucose levels were measured before injection of ^18^F-FDG, showing serum glucose levels of <150 mg/dl. Median injected activity was 304 MBq (range 251–300 MBq).

^*68*^*Ga-FAPI imaging****:*** ^68^Ga-labeled FAP ligands were used in the 31 patients with gynecological tumors: FAPI-02, *n* = 2; FAPI-04, *n* = 17; FAPI-46, *n* = 12. Median injected activity was 185 MBq (range 52–325 MBq). Additionally, the following ^68^Ga-FAPI ligands were used within our second cohort consisting of 167 female patients: FAPI-02, *n* = 9; FAPI-04, *n* = 95; FAPI-46, *n* = 35; FAPI-74, *n* = 28. Radiosynthesis and labeling was performed as described previously [13–15] for all ^68^Ga-labeled FAP ligands.

### Image evaluation

The tracer uptake in all patients for ^68^Ga-FAPI and ^18^F-FDG was quantified by mean and maximum standardized uptake values (SUVmean and SUVmax). For SUV calculation, circular volumes of interest were drawn around tumor lesions on transaxial slices and automatically adapted to a 3-dimensional volume of interest (VOI) with e.soft software (Siemens) at a 60% isocontour. Evaluation of normal organs was conducted with a 1-cm diameter (for the small organs thyroid, parotid gland, myocardium, oral mucosa, spinal cord, ovary) or 2-cm diameter (brain, muscle, liver, pancreas, spleen, kidney, fat, aortic lumen content, lung, mamma, endometrium) sphere placed inside the organ parenchyma. Tumor-to-background ratios (TBRs) were calculated for the quantification of image contrast. The geometric mean of the quotients of the lesion (SUVmax) to background tissue (SUVmean) generated the TBR, calculated for metastases in lymph nodes (relative to fat tissue), bone (relative to bone spongiosa), liver (relative to liver parenchyma), and lung (relative to lung parenchyma). Furthermore, TBRs of all present tumors were analyzed in relation to blood pool, muscle, and fat tissue. Primary lesions and metastases were defined by different factors including imaging-based diagnosis, clinical diagnosis, or histopathological confirmation. Furthermore, in 29 out of 31 patients, the tumor was biopsy proven. The ^68^Ga-FAPI-PET/CT scans were analyzed in consensus by a board-certified radiologist, a board-certified radiation oncologist, and two board-certified nuclear medicine physicians.

### FAP immunohistochemistry

Three out of the 31 patients undergoing ^68^Ga-FAPI-PET/CT provided sufficient material for the conduction of immunohistochemistry. All slices were obtained by the Tissue Bank of the National Center for Tumor Diseases (NCT) Heidelberg, Germany in concordance with the regulations of the tissue bank and the approval of the ethics committee of Heidelberg University. Immunhistochemistry was performed on 0.5-μm-thick formalin-fixed, paraffin-embedded (FFPE) tissue sections. As primary anti-FAP antibody, we utilized ab207178 (EBR20021; Abcam, Cambridge, UK), diluted 1:200. The slices were pretreated with the Buffer Substrate DAKO (pH 9, 10x, S2375) at 95° for 20 min, followed by incubation with the primary antibody at 4°C overnight. After that, we stained with the secondary antibody (DAKO EnVision and System-HRP labeled polymer anti-rabbit, code K4003) for 45 min at room temperature. In terms of visualization, Universal DAB Detection Kit (Dako Liquid DAB + Substrate Chromogen System, K3468) was utilized and counterstaining was conducted with Mayer’s hemalaun (Mayer’s Haemalaunsolution, Merck KGaA) for 10 s. Afterwards images were scanned and subsequently digitalized with the help of NanoZoomer S60 Digital slide scanner (Hamamatsu Photonics, Hamamatsu, Japan).

### Statistics

We performed descriptive analyses of all patients including demographic and tumor-specific characteristics. For the determination of standardized uptake values, median, arithmetic mean, standard deviation, and range were used. The correlation of FAPI uptake with tissue within or outside the tumor was determined using the geometrical mean. The Wilcoxon signed-rank test was performed to compare ^68^Ga-FAPI and ^18^F-FDG. The analysis between pre- and postmenopausal organs was conducted using the Mann-Whitney *U* test. A *p* value of <0.05 was defined as statistically significant. All statistical analyses were performed using SPSS Statistics Version 24 (IBM, Armonk, NY, USA) and Excel for Mac Version 15.41 (Microsoft, Redmond, Washington, USA).

## Results

### Study population

Our first cohort consisted of 31 female patients (median age 59.5 years) with various gynecological malignancies (Table 1). The following gynecological tumors were included: breast cancer (*n* = 14), ovarian cancer (*n* = 9), cervical cancer (*n* = 4), endometrial cancer (*n* = 2), leiomyosarcoma of the uterus (*n* = 1), and tubal cancer (*n* = 1). In 8 patients, the primary tumor was identified, and all 31 patients showed metastases (*n* = 81). The second group included 167 female patients with various malignancies (head-and-neck cancer, lung cancer, pancreatic cancer, gastrointestinal cancer, others) for analysis of FAPI uptake in the following normal hormone-responsive organs: endometrium (*n* = 128), ovary (*n* = 64), and breast (*n* = 147). The emerged deviations of analyzed patients and measured organs (endometrium, ovary, breast) are due to gynecological malignancies or nonidentification. Additionally, we categorized this group by age as premenopausal (*n* = 12; <35 years), postmenopausal (*n* = 68; <65 years), and unknown menstrual status (35–65 years; *n* = 87).

**Table 1:** Patient characteristics

Diagnosis	*n*	Median age	MBq	Mean SUVmax
Breast cancer	14	59	238	8.45
Ovarian cancer	9	60	236	9.29
Cervical cancer	4	57	239	15.22
Endometrial cancer	2	67	222,5	18.44
Leiomyosarcoma of the uterus	1	60	238	5.12
Tubal cancer	1	51	255	11.97

### Biodistribution in normal organs

**Fig. 1:**
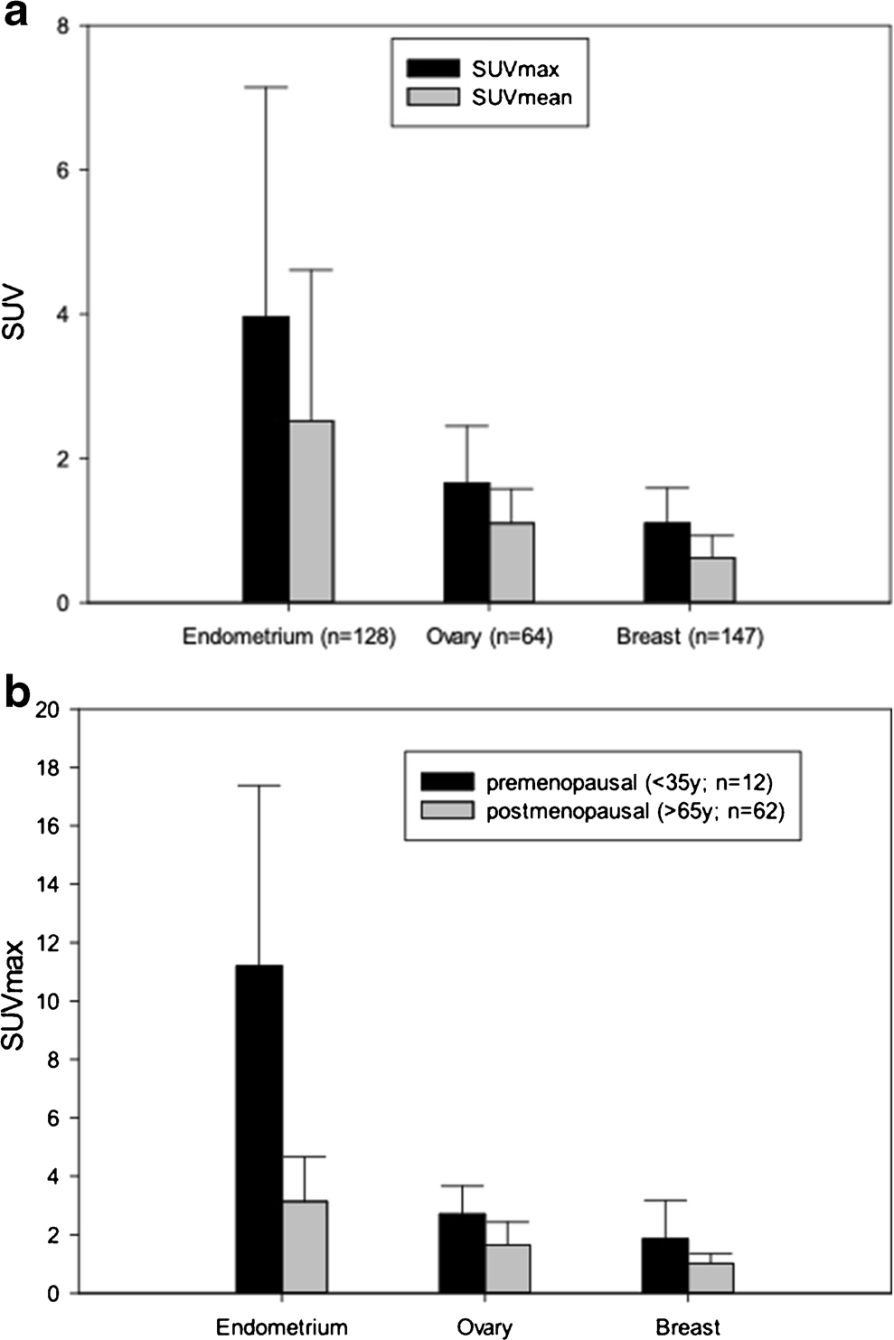
**a** PET-based evaluation of the endometrium, breast, and ovary in 167 female patients with ^68^Ga-FAPI PET/CT including various malignancies. **b** Comparison of FAPI-uptake in endometrium, ovary, and breast premenopausal (*n* = 12) and postmenopausal (*n* = 68), categorized by age

The biodistribution of ^68^Ga-FAPI in normal organs for all 31 patients with gynecological tumors is depicted in Supplement Fig. 1, showing low background activity with a mean uptake of 1.2 (SUVmax) and 0.8 (SUVmean). With regard to the uptake of ^68^Ga-FAPI and ^18^F-FDG in normal tissues in selected cases (*n* = 10 patients), mean SUVmax of FAPI was significantly lower in most normal organs (12 out of 16). Mean SUVmax showed significantly lower uptake for ^68^Ga-FAPI than ^18^F-FDG in brain parenchyma (^68^Ga-FAPI vs. ^18^F-FDG: 0.1 vs. 10.8; *p* = 0.005), oral mucosa (1.9 vs. 2.8; *p* = 0.028), parotid gland (1.4 vs. 2.0; *p* = 0.044), myocardium (1.5 vs. 3.2;; *p* = 0.017), blood-pool (mean SUVmax 1.8 vs. 2.3; *p* = 0.009), liver (1.3 vs. 3.0; p = 0.005), pancreas (1.4 vs. 2.0; *p* = 0.021), spleen (1.4 vs. 2.5; *p* = 0.012), kidney cortex (2.1 vs. 2.7; *p* = 0.007), gastrointestinal tract (measured in colon transversum: 1.3 vs 2.0; *p* = 0.008), spinal canal (0.7 vs 1.0; *p* = 0.028), and bone tissue (1.1 vs 2.3; p = 0.028). In contrast, mean SUVmax was significantly lower for ^18^F-FDG than ^68^Ga-FAPI in skeletal muscle (1.4 vs 1.0; *p* = 0.009). No significant differences were observed in normal thyroid tissue (^68^Ga-FAPI vs ^18^F-FDG: 1.8 vs 1.8; *p* = 0.878), lung parenchyma (0.8 vs 0.7; *p* = 0.507), and fat tissue (0.4 vs 0.3; *p* = 0.400). Furthermore, FAPI uptake in 167 female patients of normal hormone-responsive organs showed a mean SUVmax of 4.0 (± 3.2) in endometrium (*n* = 128), 1.7 (± 0.8) in ovary (*n* = 64), and 1.1 (± 0.5) in breast tissue (*n* = 147) as presented in Fig. 1a. Deviations of the numbers of all patients compared to the amount of ultimately measured endometrium, ovary, and breast tissues are due to gynecological malignancies or non-identification.

### Uptake in normal organs pre- and postmenopausal

FAPI uptake in normal hormone-responsive organs determined significant differences in terms of pre- and postmenopausal status. However, the FAPI uptake of the 87 female patients with unknown menstrual status was not evaluated. The analysis of 12 premenopausal (<35 years) and 68 postmenopausal (>65 years) patients showed significantly higher mean SUVmax uptake premenopausal than postmenopausal in endometrium (11.7 vs 3.0; *p* < 0.001) and breast (1.8 vs 1.0; *p* = 0.004). In contrast, FAPI accumulation in the ovaries presented no statistically significant differences pre- and postmenopausal (2.8 vs 1.6; *p* = 0.14) (Fig. 1b).

### Uptake in tumor lesions

**Fig. 2:**
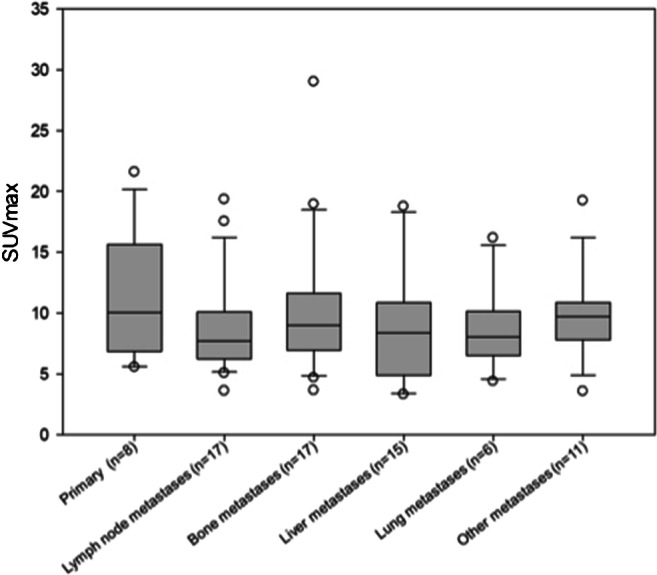
FAPI uptake in primary and metastatic lesions in 31 female patients with different gynecological tumors

Eight out of the 31 patients showed primary lesions and metastases (*n* = 81) were detected in all patients (Fig. 2). Primary lesions included primary tumors (*n* = 3), which presented a mean SUVmax and SUVmean of 10.3 (range 4.6–14.4) and 4.9 (2.8–6.8) as well as local relapses (*n* = 5) with a mean SUVmax and SUVmean of 12.3 (range 5.6–21.6) and 7.0 (range 2.7–13.1). All 81 metastases achieved a mean SUVmax and SUVmean of 10.0 (range 3.9–29.0) and 5.2 (range 2.1–10.2). The highest uptake was obtained in bone metastases (*n* = 17) with a mean SUVmax and SUVmean of 10.5 (range 3.6–29.0) and 5.4 (range 1.7–15.4). Regarding the different involved tumor entities, the strongest uptake, including all primary and secondary lesions, was measured within endometrial carcinoma with a mean SUVmax of 18.4, followed by cervical carcinoma (mean SUVmax 15.2). Thereafter, the mean SUVmax of tubal carcinoma (12.0), ovarian carcinoma (9.3), breast carcinoma (8.4), and leiomyosarcoma of the uterus (5.1) is enqueued. Comparing mean SUVmax in all metastatic lesions of ^68^Ga-FAPI-PET/CT to (^18^F-FDG) PET/CT, accumulation of FAPI shows slight advantages over FDG (8.2 vs 7.8; *p* = 0.131). Furthermore, mean SUVmax presented favorable uptake of FAPI over FDG in lymph node metastases (7.1 vs 6.3; *p* = 0.753), bone lesions (10.1 vs 7.4; *p* = 0.138), liver metastases (5.9 vs 5.1; *p* = 0.593), and significantly other metastases (10.4 vs 6.6; *p* = 0.043). Nevertheless, FDG presented a higher uptake than FAPI in lung metastases (13.7 vs 6.6; *p* = 0.18).

### Tumor-to-background ratios

**Fig. 3:**
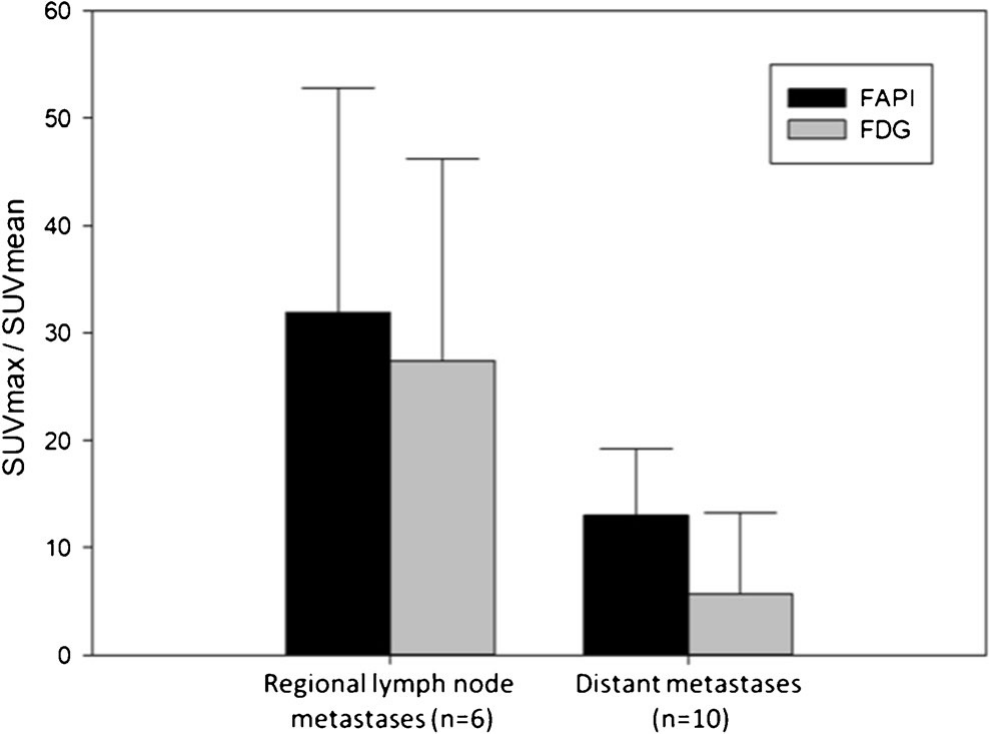
TBRs of regional lymph node metastases and distant metastases by comparing ^68^Ga-FAPI-PET/CT and ^18^F-FDG-PET/CT

Primary tumor ratios with regard to background tissue of all gynecological patients presented excellent contrasts with high geometrical means (tumor/fat 38.5; tumor/muscle 11.7; tumor/blood pool 7.6). In addition, favorable tumor-to-background ratios of 36.1 in lymph node metastases-to-fatty tissue, 17.7 in bone metastases-to-bone tissue, 23.6 in lung metastases-to-lung tissue, and 9.4 in liver metastases-to-liver tissue were determined (Supplement Table 2). Even relative to FDG, FAPI still presented slightly advantageous TBRs in regional lymph node metastases (31.9 vs 27.4; *p* = 0.6) and significantly in distant metastases (13.0 vs 5.7; *p* = 0.047), depicted in Fig. 3.

### Histology and status of BRCA1/2 mutation

**Fig. 4:**
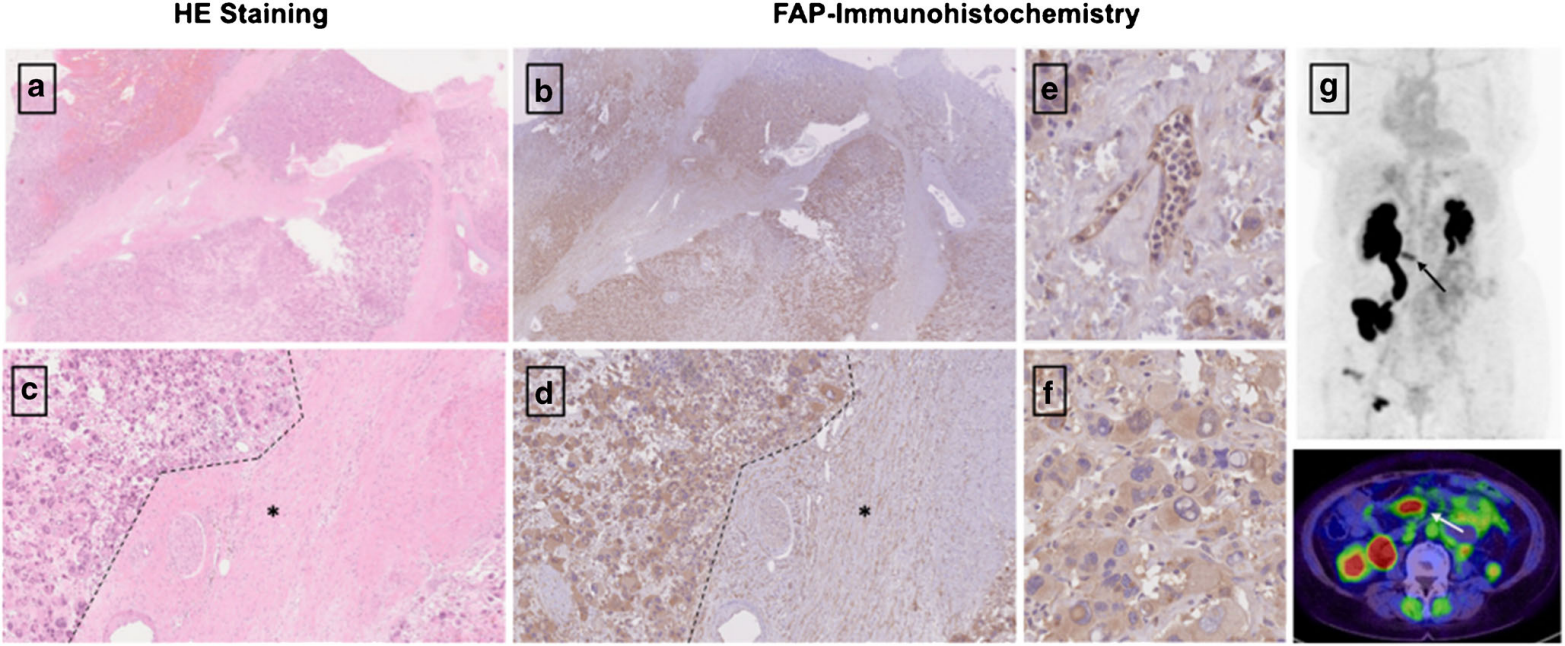
HE staining (**a**, **c**) and FAP immunohistochemistry (**b**,**c**) of a retroperitoneal metastasis in a patient diagnosed with leiomyosarcoma of the uterus. The neoplastic cells demonstrated high FAP expression [F], while normal moderate expression was depicted by the stroma (**d**). As the tumor was characterized by a high amount of angiogenesis beforehand, an example is shown in **e**, demonstrating strong FAP expression, too. [* presents stroma; --- presents neoplastic cells]. The patient presented a high SUVmax of 9.1 in ^68^Ga-FAPI-PET/CT [G], 2 days prior to the biopsy, as well

**Fig. 5:**
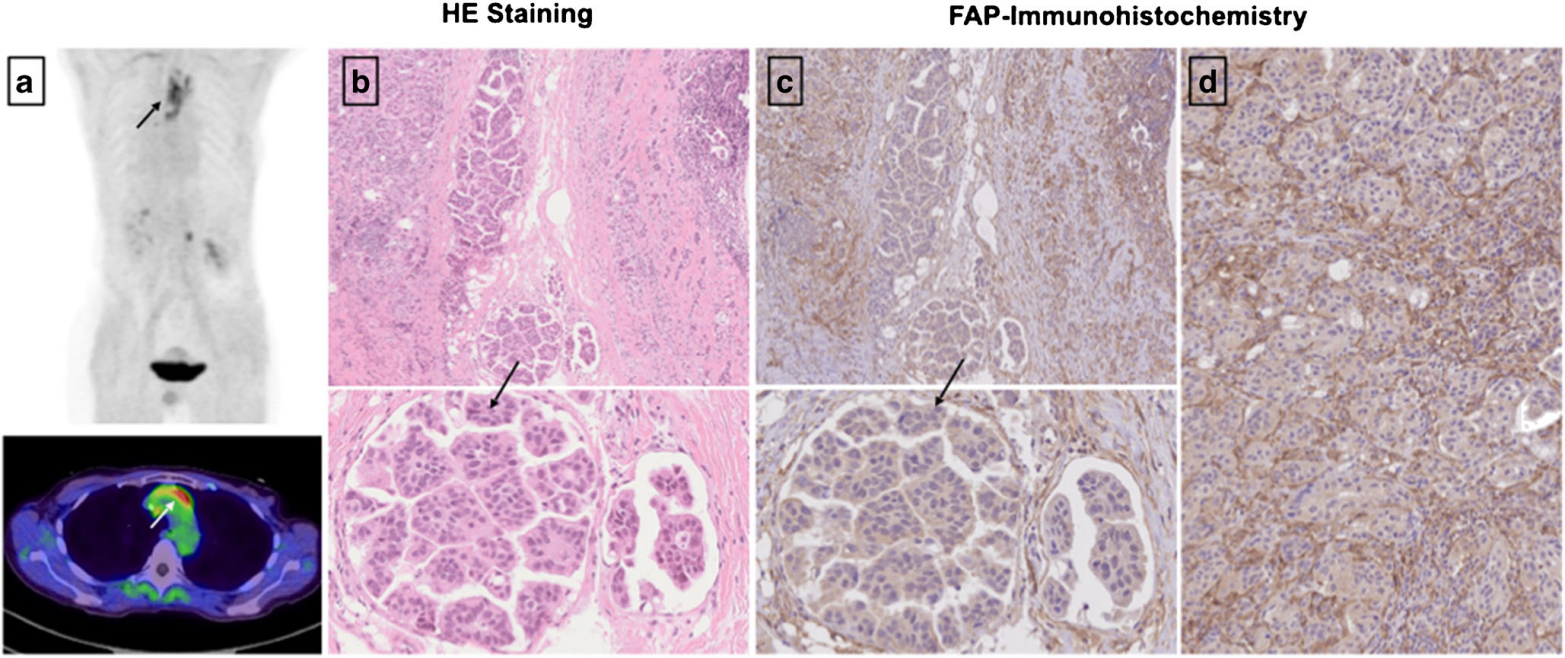
Exemplary staining with HE and anti-FAP α monoclonal antibody of a pleural biopsy due to breast cancer. The pleural metastasis, located in the mediastinum, presented a SUVmax of 7.46 in the FAPI-PET/CT 8 months prior to the biopsy (**a**). The stroma demonstrated markedly strong FAP expression (**d**), and high-to-moderate expression was observed in neoplastic cells. The depicted cell clusters (**b**, **c**) most likely represent tumorous cell nests and demonstrated strong FAP expression as well

Dividing these patients in the three groups BRCA1/2 positive (*n* = 6), BRCA 1/2 negative (*n* = 6) and unknown (*n* = 19) status, a slightly higher mean uptake was observed in BRCA1/2-positive patients than in negative patients with regard to all lesions (SUVmax 8.8 (*n* = 10) vs 7.3 (*n* = 11); *p* = 0.878). With respect to histological classification subsumed as high-grade (*n* = 9), low-grade (*n* = 8), and unknown (*n* = 14), high-grade malignancies presented a stronger uptake with a mean SUVmax of 10.3 compared to low-grade with a mean SUVmax of 8.6 (*p* = 0.266). For further characterization of FAP as a target, immunohistochemistry with anti-FAPα monoclonal antibody was exemplarily performed in three patients. Markedly strong FAP expression in terms of malignant cells was determined in two patients diagnosed with epithelial ovarian cancer and leiomyosarcoma of the uterus, which also presented FAP expression within the stroma and angioinvasion (Fig. 4). The specimen of ovarian cancer demonstrated high expression with regard to neoplastic cells, moderate expression in the stroma, and scarcely none in presumably healthy tissue allowing for precise differentiation. The third specimen, which was performed on a pleural metastasis due to breast cancer, depicted strong FAP expression within the stroma and strong-to-moderate FAP staining of the neoplastic cells. The illustrated cell clusters most likely represented tumorous cell nests which also revealed markedly high FAP expression (Fig. 5).

## Discussion

This retrospective dual-center analysis sought to evaluate the benefit and impact of ^68^Ga-FAPI-PET/CT in a small cohort of patients harboring several gynecological malignancies. Currently, gynecological imaging is performed using different diagnostic techniques, each presenting some favorable points. However, according to our results, ^68^Ga-FAPI-PET/CT seems to provide an enhancement to standard diagnostic imaging.

**Fig. 6:**
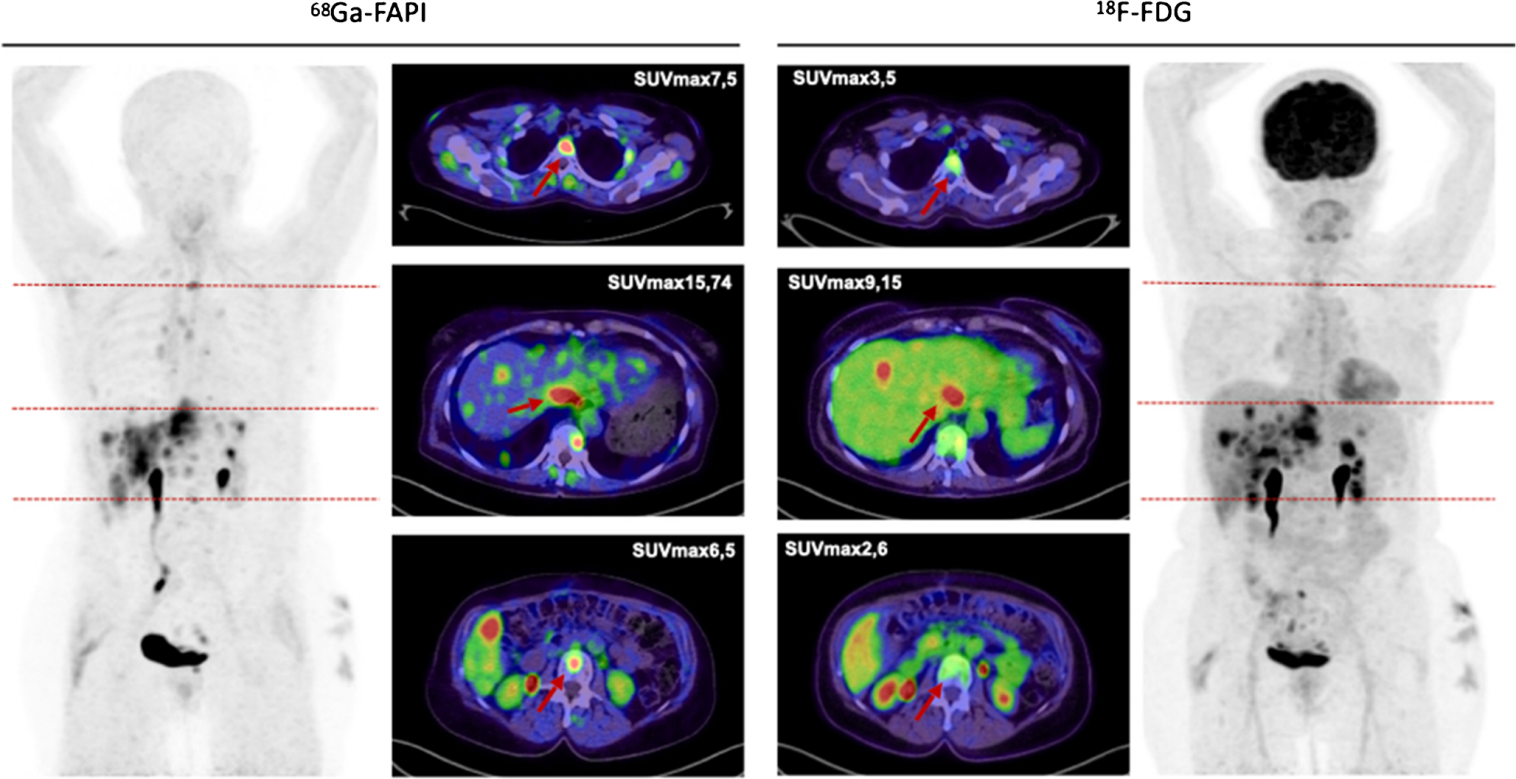
A 63-year-old woman with metastasized ovarian carcinoma underwent ^68^Ga-FAPI-PET/CT followed by ^18^F-FDG-PET/CT 1 month later. Tracer uptake in normal liver parenchyma was markedly different in both tracers: ^68^Ga-FAPI SUVmax 1.38 vs. ^18^F-FDG SUVmax 4.34. The quantified uptake in two bone metastases and one liver metastasis presented a rather high FAPI-uptake compared to FDG, respectively

**Fig. 7:**
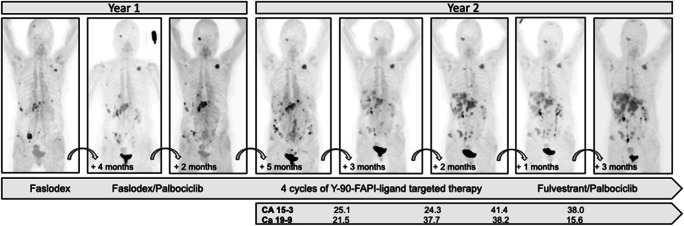
A 79-year-old female with metastasized breast and colon cancer underwent within 1.5 years eight ^68^Ga-FAPI-PET/CT with three different derivates (FAPI-02, FAPI-04, FAPI-46). During that time interval, mainly palbociclib and four cycles of Y-90-FAPI-radioligand therapy were applied. The progression markers CA 15-3 and CA 19-9 were monitored showing a temporary stable and even regredient disease, which is in concordance with FAPI uptake presented in the PET/CT. However, after a while, the patient possibly developed a resistance against palbociclib or radioligand therapy, presenting with possible progressive disease leading to a change of therapy

**Fig. 8:**
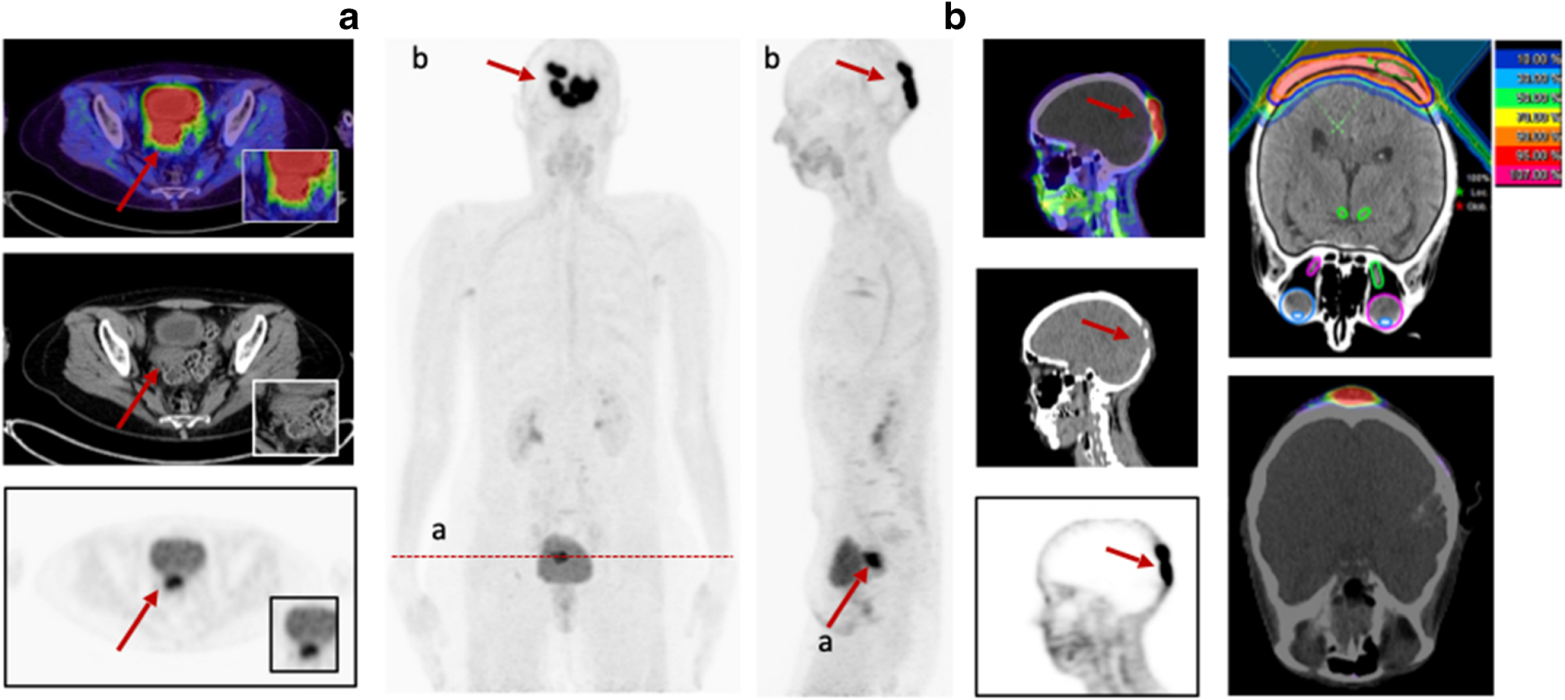
A 58-year-old female patient presented with cervical cancer for pre-radiotherapeutic staging due to a skull metastasis using ^68^Ga-FAPI-PET/CT. The investigation showed a local relapse with a SUVmax of 14.4. Furthermore, the skull metastasis presented a strong FAPI uptake with a SUVmax of 32.3 enabling precise delineation and radiotherapy planning

Biodistribution presented rather low ^68^Ga-FAPI uptake in normal organ parenchyma (Supplement Table 3), which is in concordance with previously conducted studies [13–15]. When compared to ^18^F-FDG, it achieved significantly lower or similar mean SUVmax in most normal organs with the exception of skeletal muscle. Due to very specific and high tracer uptake in metastatic lesions and low background activity, higher TBRs for ^68^Ga-FAPI-PET/CT than ^18^F-FDG, regarding regional lymph node and distant metastases, were observed. In our cohort of 31 gynecological patients, we determined very high TBRs and higher uptakes for FAPI than FDG in the lymph node, bone, liver, and other metastases. Hence, high-contrast images are established, facilitating adjacent tumor delineations and detection (Fig. 6). However, lung metastases showed a higher uptake in ^18^F-FDG-PET/CT compared to ^68^Ga-FAPI-PET/CT. Due to the very limited number of patients with lung metastases (*n* = 2), a definitive explanation is challenging to derive. Nevertheless, recent studies confirm that FAP imaging is comparable or even superior to FDG imaging in lung metastatic tumor spread [10]. Nonetheless, a supplementation or even potential solution might be provided by this novel class of radiotracers, when FDG is of limited usefulness because of inconclusive findings. Moreover, false-positive results such as increased activity in benign conditions as uterine fibroids and benign endometriotic cysts and false-negative results such as low-uptake by necrotic, cystic, or low-grade tumors can potentially be avoided [16]. In addition, early response evaluation during and after therapy is possible with ^68^Ga-FAPI-PET/CT scans, as FAP is part of the tumor microenvironment and molecular changes in the tumor stroma can subsequently be depicted (Fig. 7). Consequently, these scans may allow precise monitoring, for example, after radiotherapy, which improves staging and further radiotherapy planning (Fig. 8) [16].

However, within the comparison of ^68^Ga-FAPI and ^18^F-FDG, radiation burden and practical issues should not be neglected despite its nonspecific analysis in this study. Primarily, FAPI is independent from resting or fasting in contrast to ^18^F-FDG, whose accumulation is influenced by movement, nutrition, and blood glucose levels. Yet, further features of a FAPI-PET/CT scan are presented by its feasibility for quick image acquisition with adequate images 10 min postinjection, its fast clearance, and its mostly lower off-target accumulation in comparison to FDG [9]. As a result, possibly reduced radiation doses, even when compared to (^18^F-FDG) PET/CT, as well as improved practical implementation can be observed [9, 13, 14, 17]. Remarkably, FAPI, a single molecule, is both a diagnostic and possibly therapeutic agent, enabling additional theranostic application.

In the second analyzed cohort of 167 female patients with different tumor entities, normal hormone-responsive organs presented rather high (endometrium) and intermediate (breast, ovary) FAPI uptake. Regarding these patients, a high standard deviation of all measured SUVmax in the endometrium (3.2) and rather moderate standard deviations in the ovary (0.8) and breast (0.5) were determined. This observation indicates that changes in hormone response might influence FAPI uptake to a different extent in these organs. Supplementary, the highest uptake was shown by the endometrium with a mean SUVmax of 4.0, while ovary and breast showed moderate uptake with 1.7 and 1.1, respectively. A potential explanation is a correlation with the presence or absence of the menstrual cycle. To explore this more closely, we compared FAPI uptake in pre- and postmenopausal women determining significantly higher uptakes premenopausal in the endometrium and breast, while no significant differences were observed in the ovaries. The evaluated significantly higher premenopausal uptake of the endometrium might be due to cyclic regeneration as FAPI is known to accumulate in tissue during remodeling processes [5]. This observation might be substantially relevant for precise interpretation of PET/CT images, as increased FAPI uptake of the endometrium may not necessarily indicate infiltration of the tumor into the uterus. Nevertheless, postmenopausal FAPI uptake was relevant, proposing that the postmenopausal endometrium is still active and rather in a quiescent state than truly atrophic [18].

Previously conducted studies indicated that differences in breast parenchyma are based on dynamic physiologic processes of the inner environment in response to endogenous and exogenous factors such as increased or decreased hormonal stimulations [19]. Our analysis revealed low uptake in normal breast tissue, whilst a rather high mean SUVmax of 8.45 was observed in breast cancer. Analyses of histopathology suggested that strong-to-moderate FAP expression is present in the stroma of breast carcinomas [20, 21]. Furthermore, breast density, evaluated by computer tomography, seems to decrease as age increases for both pre- and postmenopausal women, particularly during the menopausal transition [22–24]. On the contrary, pregnancy and lactation increase breast density as hormonal stimulation changes [25]. Similarly, FAPI uptake seems to be increased as well in hormone-responsive organs such as endometrium and breast during lactation and after hormonal stimulation, observed in two individual case reports [26, 27]. As significant differences pre- and postmenopausal were shown, we hypothesize that modifications regarding cellular FAP expression or the number of FAP-positive fibroblasts might be a consequence of hormonal changes and therefore a differently stimulated inner environment.

**Fig. 9:**
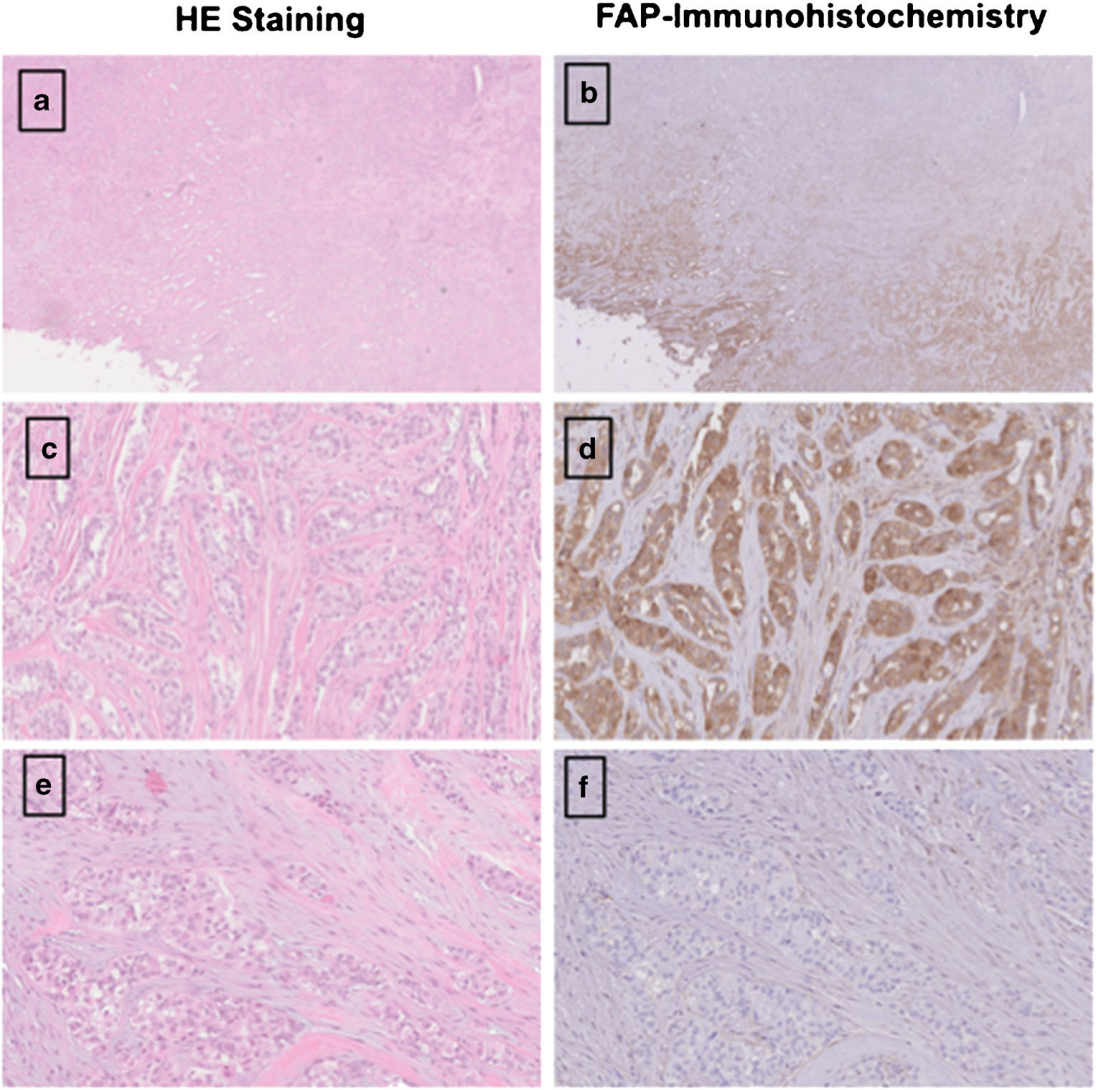
HE Staining (**a**) and FAP immunohistochemistry (**b**) of a high-grade endometrial ovarian adenocarcinoma. Remarkably, the malignant cells (**c**, **d**) demonstrated very strong FAP expression (**d**) compared to presumably uninfiltrated tissue which showed with exception of the stroma scarcely none (**e**, **f**) facilitating differentiation between healthy and malignant tissue

**Fig. 10:**
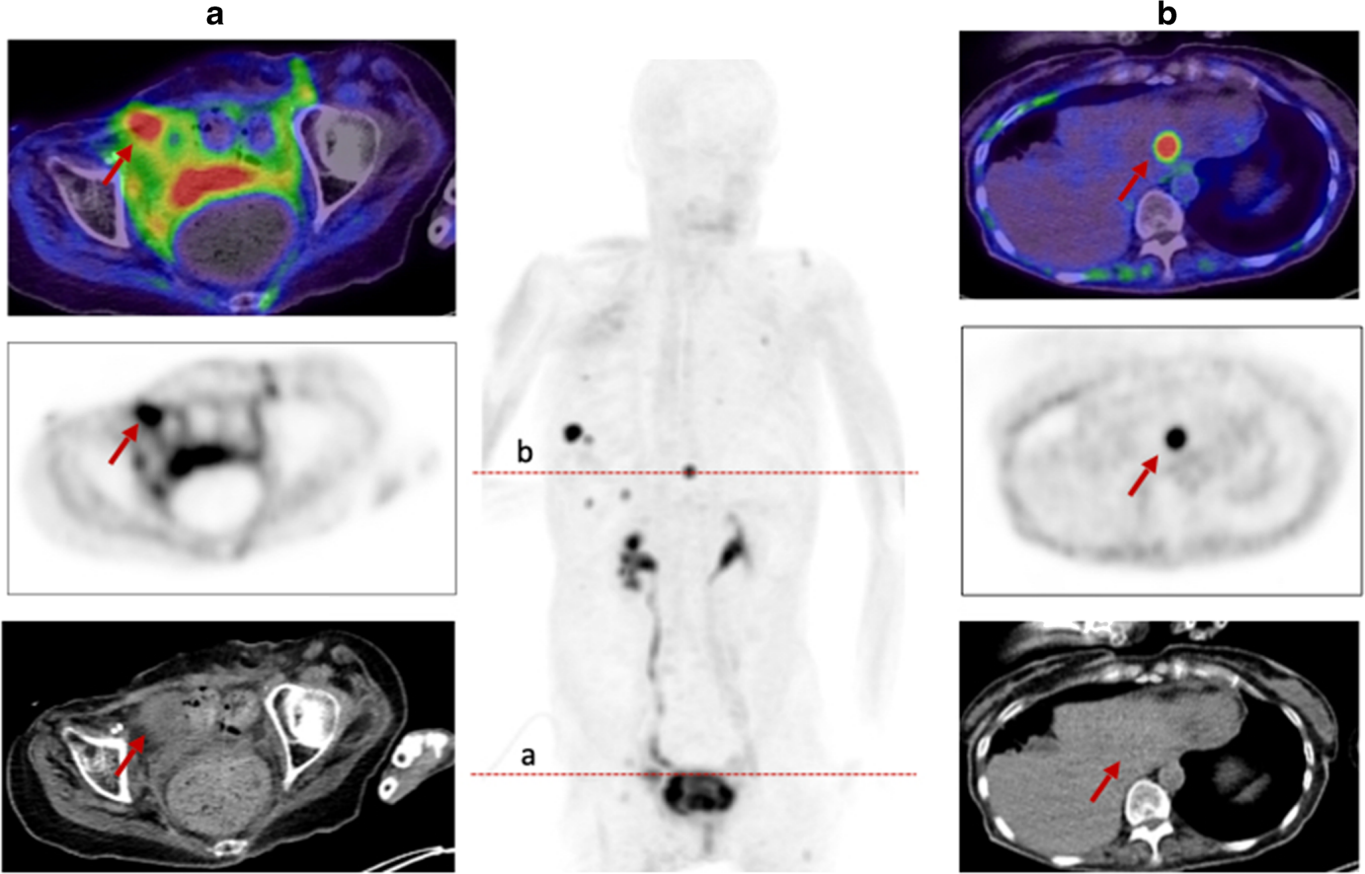
A 60-year-old patient with metastasized cervical carcinoma underwent ^68^Ga-FAPI-PET/CT due to monitoring. The local relapse presented a rather strong FAPI uptake with SUVmax 16.90, similar to the exemplary shown liver metastasis in segment II (SUVmax 14.1)

The current imaging findings are partially in concordance with previous histopathology studies, which presented intermediate expression of FAP in endometrial cancer, cervix cancer, and ovarian cancer and high expression in breast cancer [8, 28]. Ovarian cancer was well in line with these studies as it showed intermediate uptake. Remarkably, exemplary immunohistochemistry of a patient with high-grade endometrial ovarian cancer demonstrated moderate FAP expression in the stroma, markedly strong FAP expression within neoplastic cells, and scarcely none in presumably healthy non-tumorous cells, enabling a clear distinction between malignant and healthy tissue (Fig. 9). Therefore, targeting FAP might present a potential pleiotropic antitumor strategy for epithelial ovarian cancer, as a high expression of FAP in CAFs has proven to influence proliferation, invasion, therapy resistance, shorter recurrence, and poor clinical outcome [29–32]. Furthermore, limited therapeutic strategies are existent, chemoresistance is rather frequently developed, and FAP proved to be absent in normal ovaries [31]. In summary, further investigations are inevitably of essence to hopefully overcome some of these challenges. In endometrial and cervix cancer, the highest uptake was measured, compared to intermediate staining. Hence, it justifies that imaging should be added to the workup and treatment planning of patients with cervical cancer when available, since it provides significant additional information to determine TNM stage and the best choice of treatment, such as the relationship between primary cancer and adjacent tissue, lymph node involvement, and distant metastases [33, 34]. Furthermore, dispelling the fact that staging of uterine cervical cancer should not be based on nonimaging clinical parameters, determined by the FIGO classification, due to the fact that cervical cancer is the most common female malignancy in the developing countries (Fig. 10) [34].

In patients with breast cancer, the lesions presented a moderate uptake in relation to a rather high expression of FAP [20, 21]. Within our exemplary staining of a pleural biopsy in a patient diagnosed with breast cancer, very high FAP expression in the stroma and strong-to-moderate expression in the neoplastic cells and tumor cell nests were observed as well. Therefore, these deviations might be due to low patient numbers and low amounts of present primary tumors in our analysis. Moreover, high FAP expression in breast and ovarian cancer seems to correlate with advanced tumor grades and worse prognosis [35–38]. This is in line with our results, as FAPI uptake, with respect to histological classification, presented to be stronger in high-grade than low-grade lesions (Supplement Fig. 2). Furthermore, uptake seems to correlate with the status of BRCA1/2 mutation. Within patients presenting a BRCA1/2 mutation, slightly higher uptakes were determined as compared to patients without mutation.

Our retrospective analysis contained several limitations such as the use of different FAPI ligands; nonetheless, these derivates share a common backbone, and thus early-phase biodistribution and tumor uptake seem to be still comparable regarding diagnostic evaluation. However, FAPI-04 and FAPI-46 are indeed characterized by longer tumor retention time 1 h p.i., compared with FAPI-02, yet this aspect is solely relevant for FAP-targeted radionuclide therapy and hence not of essence in this analysis [13–15]. Due to the limited number of patients, no subgroup analyses regarding a comparison with ^18^F-FDG as well as correlation with histologic classification and BRCA1/2 mutation status for the same tumor type were possible. The latter, combined with a limited amount of FAPI and FDG scans, highlights the inevitable necessity for more precise analyses of patients with gynecological malignancies in order to evaluate the significance of our theses. Physiological endometrial FAPI uptake may also vary during the menstrual cycle, which limits our analyses, as evaluation of FAPI uptake in correlation with different menstrual phases was beyond the scope of this work. Additionally, only 29 out of 31 tumors were biopsy proven, representing a limitation of this study. Further research regarding this topic is of essence, as it may assist in avoiding false-positive interpretations and findings.

## Conclusion

^68^Ga-FAPI-PET/CT crystalizes as a highly promising tracer regarding staging and follow-up of gynecological malignancies, as it achieved high tracer uptake, sharp contrasts in primary and secondary lesions, and in addition, higher TBRs than ^18^F-FDG-PET/CT. Furthermore, the inner environment or normal hormone-responsive organs seem to influence FAPI uptake which is essential for precise and accurate interpretation of ^68^Ga-FAPI-PET/CT scans. Larger prospective studies will have to establish the precise sensitivity, specificity, and accuracy of ^68^Ga-FAPI in gynecological malignancies as well as the influence of external and internal hormonal stimulation on FAPI uptake.

## Supplementary information

ESM 1 (JPEG 61 kb)

ESM 2 (JPEG 79 kb)

ESM 3 (DOCX 15 kb)

ESM 4 (JPEG 59 kb)

ESM 5 (JPEG 64 kb)

## Data Availability

The data used and/or analyzed during the current study are available from the corresponding author on reasonable request.
