# Supplementary figures and images for: 68Ga-FAPI-PET/CT in patients with various gynecological malignancies
Source: Eur J Nucl Med Mol Imaging. 2021 May 29;48(12):4089–100. doi: 10.1007/s00259-021-05378-0 (PMC8484099; doi:10.1007/s00259-021-05378-0)

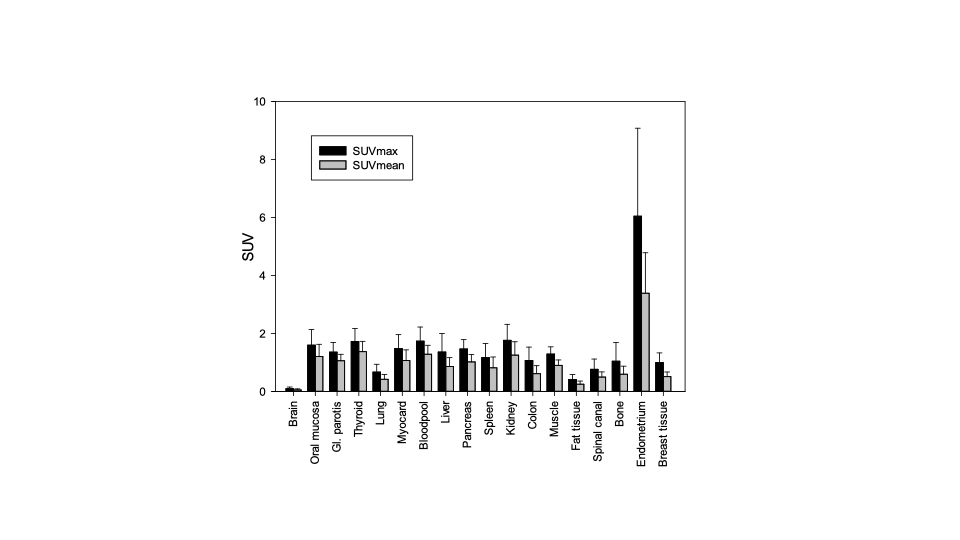

Supplement: Supplementary file 1 — (JPEG 61 kb) [file 259_2021_5378_MOESM1_ESM.jpeg]

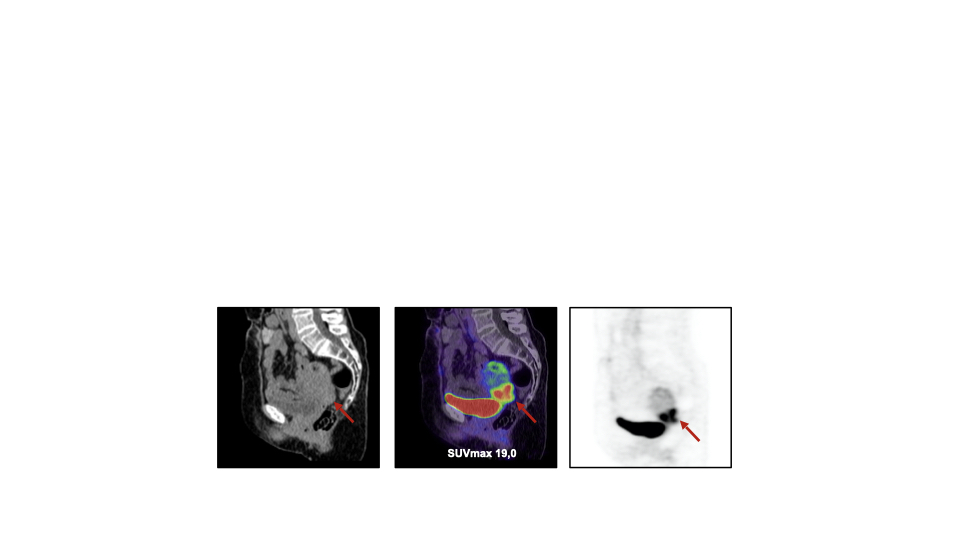

Supplement: Supplementary file 2 — (JPEG 79 kb) [file 259_2021_5378_MOESM2_ESM.jpeg]

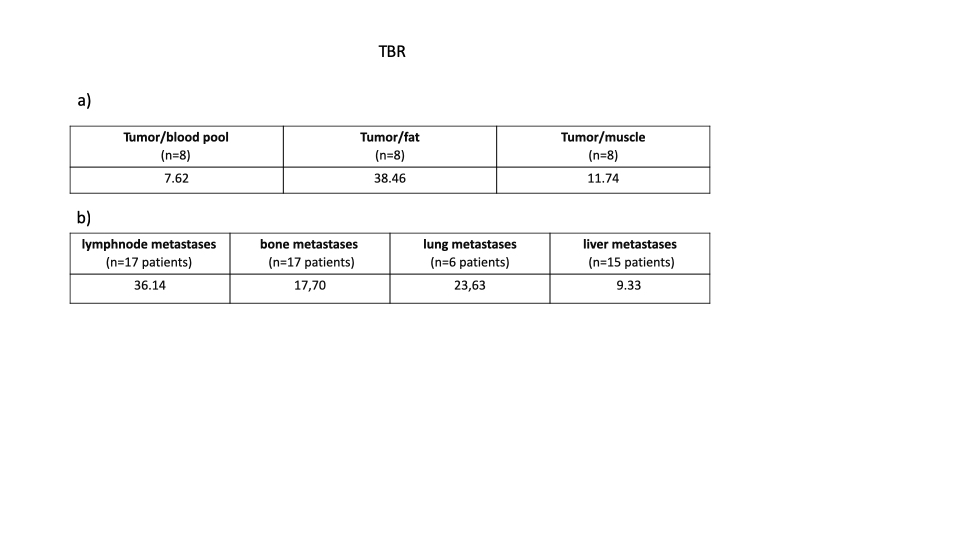

Supplement: Supplementary file 4 — (JPEG 59 kb) [file 259_2021_5378_MOESM4_ESM.jpeg]

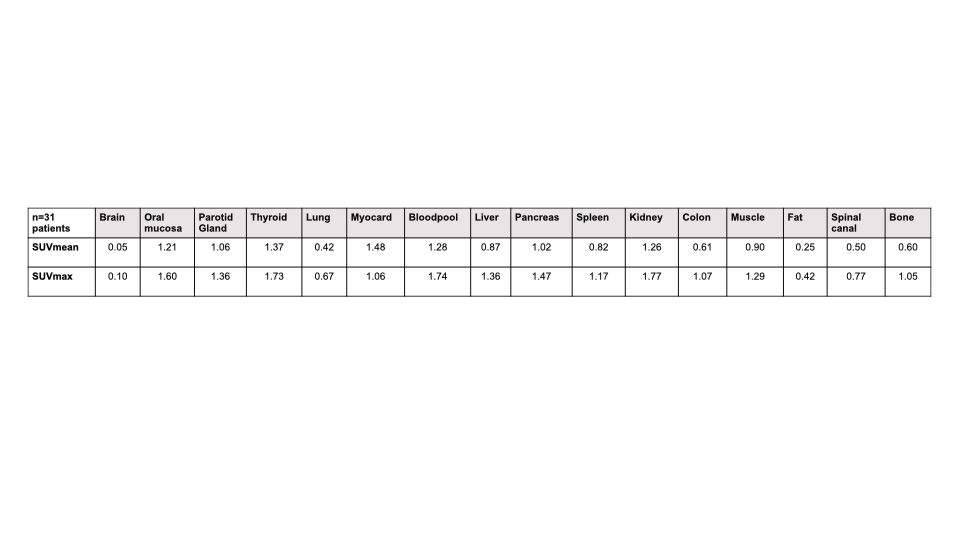

Supplement: Supplementary file 5 — (JPEG 64 kb) [file 259_2021_5378_MOESM5_ESM.jpeg]
