# Supplementary material for: 68Ga-FAPI-PET/CT in patients with various gynecological malignancies
Source: Eur J Nucl Med Mol Imaging. 2021 May 29;48(12):4089–100. doi: 10.1007/s00259-021-05378-0 (PMC8484099; doi:10.1007/s00259-021-05378-0)
Supplement: Supplementary file 3 — (DOCX 15 kb) [file 259_2021_5378_MOESM3_ESM.docx]

| Site | Heidelberg University Hospital | University of Pretoria |
| --- | --- | --- |
| PET/CT scanner | Biograph mCT Flow, Siemens | Biograph mCT 40 slice, Siemens |
| Injected activity (MBq), ^68^Ga-FAPI | 239 | 78 |
| Injected activity (MBq), ^18^F-FDG | 312 | 305 |
| Time interval (median days) | 9 | 1 |
| CT reference (mAs) | 30 | 40 - 150 |
| CT peak kilovoltage (keV) | 130 | 120 |
| CT slice thickness (mm) | 5 | 5 |
| CT slice increment (mm) | 3 - 4 | 5 |
| PET reconstruction | OSEM algorithm | OSEM algorithm |
| Iterations | 2 | 4 |
| Subsets | 21 | 8 |
| Matrix | 200 x 200 | 200x200 |
| Corrections | Gaussian 5 mm FWHM | Gaussian filter applied at FWHM of 5.0mm |
